# Phase I Single Ascending Dose and Food Effect Study in Healthy Adults and Phase I/IIa Multiple Ascending Dose Study in Patients with Pulmonary Tuberculosis to Assess Pharmacokinetics, Bactericidal Activity, Tolerability, and Safety of OPC-167832

**DOI:** 10.1128/aac.01477-22

**Published:** 2023-05-23

**Authors:** Rodney Dawson, Andreas H. Diacon, Kim Narunsky, Veronique R. De Jager, Kelly W. Stinson, Xiaoyan Zhang, Yongge Liu, Jeffrey Hafkin

**Affiliations:** a Division of Pulmonology, Department of Medicine, University of Cape Town and University of Cape Town Lung Institute, Cape Town, South Africa; b TASK Applied Science, Cape Town, South Africa; c Cultura, LLC, Decatur, Georgia, USA; d Otsuka Pharmaceutical Development & Commercialization, Inc., Rockville, Maryland, USA

**Keywords:** OPC-167832, decaprenylphosphoryl-β-d-ribose 2-oxidase, tuberculosis, antibacterial, safety and tolerability, pharmacokinetics, bactericidal activity, phase I study, phase I/IIa study

## Abstract

OPC-167832, an inhibitor of decaprenylphosphoryl-β-d-ribose 2’-oxidase, demonstrated potent antituberculosis activity and a favorable safety profile in preclinical studies. This report describes the first two clinical studies of OPC-167832: (i) a phase I single ascending dose (SAD) and food effects study in healthy participants; and (ii) a 14-day phase I/IIa multiple ascending dose (MAD; 3/10/30/90 mg QD) and early bactericidal activity (EBA) trial in participants with drug-susceptible pulmonary tuberculosis (TB). OPC-167832 was well tolerated at single ascending doses (10 to 480 mg) in healthy participants and multiple ascending doses (3 to 90 mg) in participants with TB. In both populations, nearly all treatment-related adverse events were mild and self-limiting, with headache and pruritus being the most common events. Abnormal electrocardiograms results were rare and clinically insignificant. In the MAD study, OPC-167832 plasma exposure increased in a less than dose-proportional manner, with mean accumulation ratios ranging from 1.26 to 1.56 for *C*_max_ and 1.55 to 2.01 for area under the concentration-time curve from 0 to 24 h (AUC_0–24h_). Mean terminal half-lives ranged from 15.1 to 23.6 h. Pharmacokinetics (PK) characteristics were comparable to healthy participants. In the food effects study, PK exposure increased by less than ~2-fold under fed conditions compared to the fasted state; minimal differences were observed between standard and high-fat meals. Once-daily OPC-167832 showed 14-day bactericidal activity from 3 mg (log_10_ CFU mean ± standard deviation change from baseline; −1.69 ± 1.15) to 90 mg (−2.08 ± 0.75), while the EBA of Rifafour e-275 was −2.79 ± 0.96. OPC-167832 demonstrated favorable pharmacokinetic and safety profiles, as well as potent EBA in participants with drug-susceptible pulmonary TB.

## INTRODUCTION

According to recent statistics from the World Health Organization, 1.6 million people died in 2021 from tuberculosis (TB), making it the second leading infectious disease killer after COVID-19 (1). The persistence of the TB pandemic is in part a consequence of the complexity, length, and toxicity of current regimens, which can reduce adherence to treatment (2). It also reflects the ongoing and serious problem of multidrug-resistant (MDR) strains of Mycobacterium tuberculosis that are no longer susceptible to first-line drugs, requiring instead more toxic second-line regimens that necessitate longer durations of treatment. Consequently, there is a dire need for new anti-TB drugs with novel mechanisms, increased potencies, better safety profiles, and shorter treatment durations to ultimately turn the tide on this enduring pandemic.

In pursuit of such novel anti-TB drugs, OPC-167832 was identified in a phenotypic screen that selected for and optimized carbostyril derivatives with anti-TB activity (3). In preclinical evaluations, OPC-167832 was shown to be highly potent, with MICs against various M. tuberculosis strains, including MDR strains, ranging from 0.00024 to 0.002 μg/mL. In a mouse model of TB, OPC-167832 demonstrated potent bactericidal activity, particularly in combination with delamanid, bedaquiline, or levofloxacin (3). In a recent study using the C3HeB/FeJ mouse model, which is believed to develop TB lung lesions resembling those in diseased humans, OPC-167832 treatment was associated with significant reductions in bacterial load (4). Nonclinical safety evaluations demonstrated that OPC-167832 had acceptable toxicologic, safety, and tolerability profiles in animal model systems (Otsuka Pharmaceuticals, data on file).

Based on these favorable results, OPC-167832 proceeded to initial human trials. First, a phase I study in healthy adults was conducted to assess the pharmacokinetic, safety, and tolerability profiles of single ascending doses of OPC-167832, as well as the effects of food on its absorption, distribution, and elimination. This phase I study was followed by a phase I/IIa study, in which adult participants with drug-susceptible pulmonary TB were treated for 14 days with ascending doses of OPC-167832 monotherapy. The latter trial was designed to assess not only the pharmacokinetic, safety, and tolerability profiles of OPC-167832, but also to provide preliminary data on early bactericidal activity (EBA) in a clinical setting. The current report describes outcomes from both trials. In addition, as a secondary objective, this report described the use of a biomarker, lipoarabinomannan (LAM) concentration in sputum as measured by a LAM-enzyme-linked immunosorbent assay (LAM-ELISA) as a guide for dose selection in the EBA trial (5).

## RESULTS

### Participants.

In part 1 of the phase I study (SAD study), 48 healthy adult males were screened and subsequently enrolled. All participants completed the trial. In part 2 (food effects study), 12 healthy adult males were screened and subsequently enrolled. One of 6 (16.7%) participants discontinued early in the standard, then fasted, then high-fat group (1 consent withdrawal during period 1). Two of 6 (33.3%) participants discontinued early in the fasted, then high-fat, then standard meal group (1 consent withdrawal in period 1, and 1 positive urine drug screen in period 2). All other participants in the two treatment sequences completed the trial. Demographic and baseline characteristics were generally similar across dose cohorts in the SAD study (see Table S1 in the supplemental material) and across the two treatment sequences in the food effects study (Table S2).

In the MAD/EBA study, 76 HIV-negative adults with smear-positive, rifampicin- and isoniazid-susceptible pulmonary TB were enrolled. Of these, 59 received OPC-167832 (3 mg [*n* = 14], 10 mg [*n* = 14], 30 mg [*n* = 14], or 90 mg [*n* = 17]). Of the remaining 17 participants, 16 were treated with RHEZ and 1 who was initially assigned to the RHEZ arm discontinued early due to prior attainment of the enrollment target. No subject in the OPC-167832 3 mg, 10 mg, or 30 mg cohort discontinued early, whereas 3/17 (17.6%) participants discontinued early in the OPC-167832 90 mg cohort due to later findings that they met exclusion criteria.

Demographic and baseline bacterial characteristics (sputum CFU/mL, sputum LAM concentrations, and MGIT-TTD) were comparable across the dosing cohorts in the MAD/EBA study (Table S3). Mean baseline log_10_ CFU/mL in sputum samples across treatment groups was >6, consistent with a population of adults with smear-positive pulmonary TB. Body mass indices were lower in the MAD/EBA study population, comprised of participants with TB, compared to the healthy population in the SAD and food effects studies.

### Pharmacokinetics.

**FIG 1:**
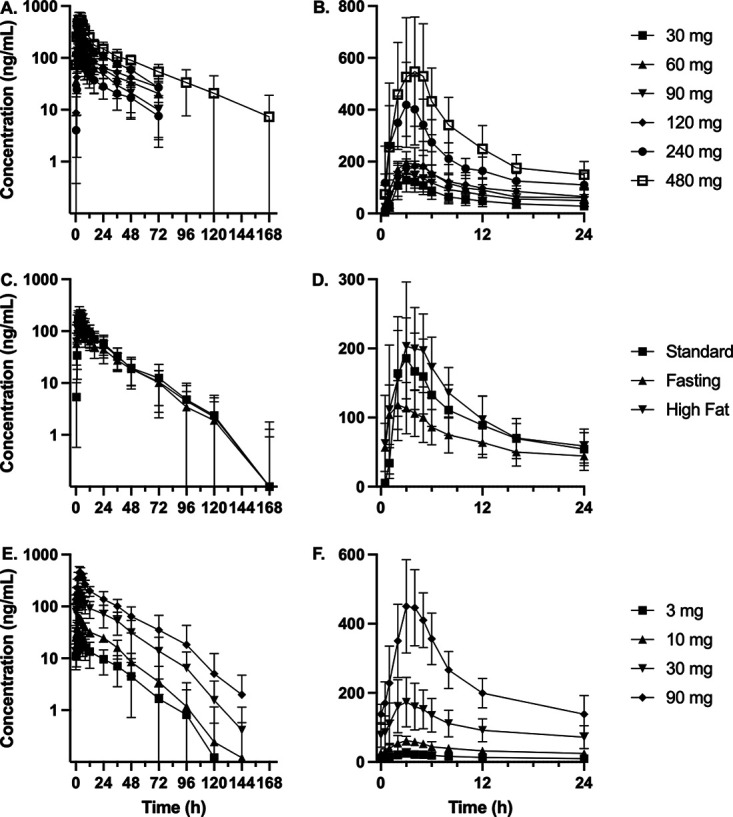
OPC-167832 pharmacokinetics. (A and B) Mean plasma concentration versus time in healthy adult participants following single administrations of OPC-167832. (C and D) Mean plasma concentration versus time in healthy adult participants following a single 60-mg dose of OPC-167832 administered after a standard meal, fasting or a high-fat meal. (E and F) Mean plasma concentrations of OPC-167832 versus time at steady state (day 14) following once-daily administrations of OPC-167832 in patients with rifampicin- and isoniazid-susceptible pulmonary TB. A, C, and E present the data on semilogarithmic plots. B, D, and F present the data on linear plots. Time points beyond 24 h are not presented in B, D, and F to clearly distinguish early time points. Error bars are ± SD.

**FIG 2:**
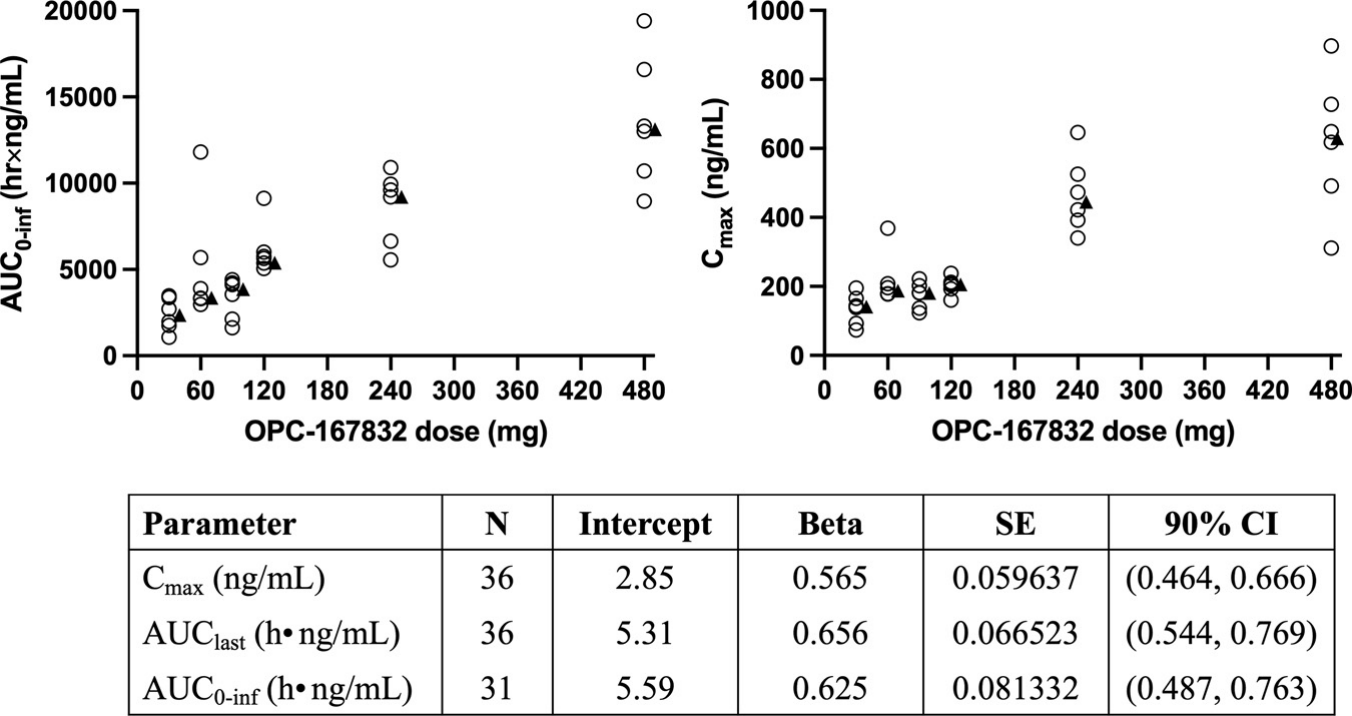
Dose proportionality of OPC-167832. AUC_0–inf_ (upper left) and *C*_max_ (upper right) versus OPC-167832 single dose level for 6 participants in each cohort in the SAD study. Summary of power model of PK endpoints on log scale (bottom).

Pharmacokinetic outcomes for the individual cohorts in the SAD study are presented in Fig. 1A and Table 1. In general, *C*_max_ and AUC_0–inf_ increased in a less than dose-proportional manner up to OPC-167832 480 mg (Fig. 2). Across dosing cohorts, median *T*_max_ occurred between 2.5 and 3.5 h after administration, and mean terminal half-lives ranged from 19.5 to 30.0 h. In the food effects study, AUC_0–inf_ and *C*_max_ increased less than ~1.5 to 2-fold in fed condition compared to administration in the fasted state, while delaying median *T*_max_ slightly from 2 to 3.5 h (Fig. 1B and Table S4). Minimal differences in exposure were observed following standard versus high-fat meals.

**TABLE 1:** Single dose pharmacokinetic parameters of OPC-167832^*a*^

Parameter, unit	OPC-167832					
	30 mg	60 mg	90 mg	120 mg	240 mg	480 mg
	*n* = 6	*n* = 6	*n* = 6	*n* = 6	*n* = 6	*n* = 6
*C*_max_, ng/mL	135 ± 45.0	218 ± 74.9	175 ± 37.6	203 ± 25.6	466 ± 109	616 ± 200
*T*_max_, h (median [range])	3.00 (2.00, 5.00)	3.00 (1.02, 5.00)	2.50 (1.00, 5.00)	3.50 (2.00, 5.00)	3.00 (1.00, 4.00)	3.50 (2.00, 5.00)
AUC_last_, h•ng/mL	2118 ± 813	4070 ± 2258	3008 ± 970	4560 ± 1276	7526 ± 1862	13,200 ± 3531
AUC_0–inf_, h•ng/mL	2392 ± 951	5421 ± 3724	3346 ± 1180	6524 ± 2264	8441 ± 2256	13,660 ± 3832
t_½_, h	22.1 ± 6.93	28.9 ± 10.2	19.5 ± 7.51	30.0 ± 3.34	21.9 ± 7.76	25.8 ± 13.0
CL/F, mL/min	247 ± 121	243 ± 94.9	517 ± 240	337 ± 61.6	491 ± 139	626 ± 175
CL/F/BW, mL/min/kg	3.48 ± 1.16	3.73 ± 1.47	6.24 ± 2.77	4.38 ± 0.82	6.17 ± 1.85	7.96 ± 2.82
*C*_max_/dose, ng/mL/mg	4.50 ± 1.50	3.64 ± 1.25	1.94 ± 0.42	1.69 ± 0.21	1.94 ± 0.45	1.28 ± 0.42
AUC_last_/dose, h•ng/mL/mg	70.6 ± 27.1	67.8 ± 37.6	33.4 ± 10.8	38.0 ± 10.6	31.4 ± 7.76	27.5 ± 7.36
AUC_0–inf_/dose, h•ng/mL/mg	79.7 ± 31.7	90.4 ± 62.1	37.2 ± 13.1	54.4 ± 18.9	35.2 ± 9.40	28.5 ± 7.98

a Values are mean ± standard deviation, unless noted. AUC_0–inf_, area under the concentration-time curve from zero to infinity; AUC_0–last_, area under the concentration-time curve from zero to last sample collection time; BW, body weight; CL/F, apparent clearance; *C*_max_, maximum (peak) plasma concentration; SD, standard deviation; t_1/2_, terminal-phase elimination half-life; *T*_max_, time to maximum (peak) plasma concentration.

Pharmacokinetic outcomes for the individual cohorts in the MAD study are presented in Fig. 1C and Table 2. In participants with drug-susceptible pulmonary TB, OPC-167832 plasma exposure also increased less than dose-proportionally, with mean accumulation ratios ranging from 1.26 to 1.56 for *C*_max_ and 1.55 to 2.01 for AUC_0–24h_. Minimal variation was observed in predose concentrations on days 12 to 14, indicating the attainment of apparent steady state by that time (data not shown). At steady state, mean terminal half-lives ranged from 15.1 to 23.6 h, and median *T*_max_ was approximately 3.0 h. A *post hoc* analysis demonstrated that steady-state OPC-167832 exposure was similar in male and female participants (data not shown).

**TABLE 2:** Multiple dose pharmacokinetics of OPC-167832^*a*^

Parameter, unit	Day 1				Day 14			
	OPC-167832				OPC-167832			
	3 mg	10 mg	30 mg	90 mg	3 mg	10 mg	30 mg	90 mg
	(*n* = 14)	(*n* = 14)	(*n* = 14)	(*n* = 17)	(*n* = 13)	(*n* = 14)	(*n* = 14)	(*n* = 14)
*C*_max_, ng/mL	20.7 ± 6.92	53.7 ± 9.16	128 ± 50.5	391 ± 116	28.2 ± 10.9	67.3 ± 11.9	190 ± 63.6	492 ± 99.8
*T*_max_, h (median [range])	3.17 (2.17, 4.17)	4.15 (1.17, 5.15)	3.15 (2.15, 5.17)	3.25 (2.13, 5.17)	3.17 (1.18, 5.17)	3.15 (1.15, 5.13)	3.13 (2.13, 5.13)	3.17 (2.13, 5.15)
AUC_0–24h_, h•ng/mL	178 ± 65.1	569 ± 102	1290 ± 532	4050 ± 1240	349 ± 143	874 ± 127	2490 ± 928	5690 ± 1010
t_1/2_, h					23.6 ± 9.3	15.4 ± 4.2	15.1 ± 6.2	15.7 ± 5.8
CL/F, mL/min					164 ± 62.6	195 ± 28.8	231 ± 91.8	272 ± 53.8
CL/F/BW, mL/min/kg					2.90 ± 0.94	3.21 ± 0.32	4.21 ± 1.33	5.15 ± 1.10
Accumulation ratio, *C*_max_					1.39 ± 0.38	1.26 ± 0.17	1.56 ± 0.40	1.36 ± 0.36
Accumulation ratio, AUC_0–24h_					1.99 ± 0.55	1.60 ± 0.29	2.01 ± 0.57	1.55 ± 0.42
*C*_max_/dose, ng/mL/mg					9.42 ± 3.63	6.73 ± 1.19	6.34 ± 2.12	5.46 ± 1.11
AUC_0–24h_/dose, h•ng/mL/mg					116 ± 47.6	87.4 ± 12.7	82.8 ± 30.9	63.3 ± 11.2

a Values are mean ± standard deviation, unless noted.

### Bactericidal activity.

**FIG 3:**
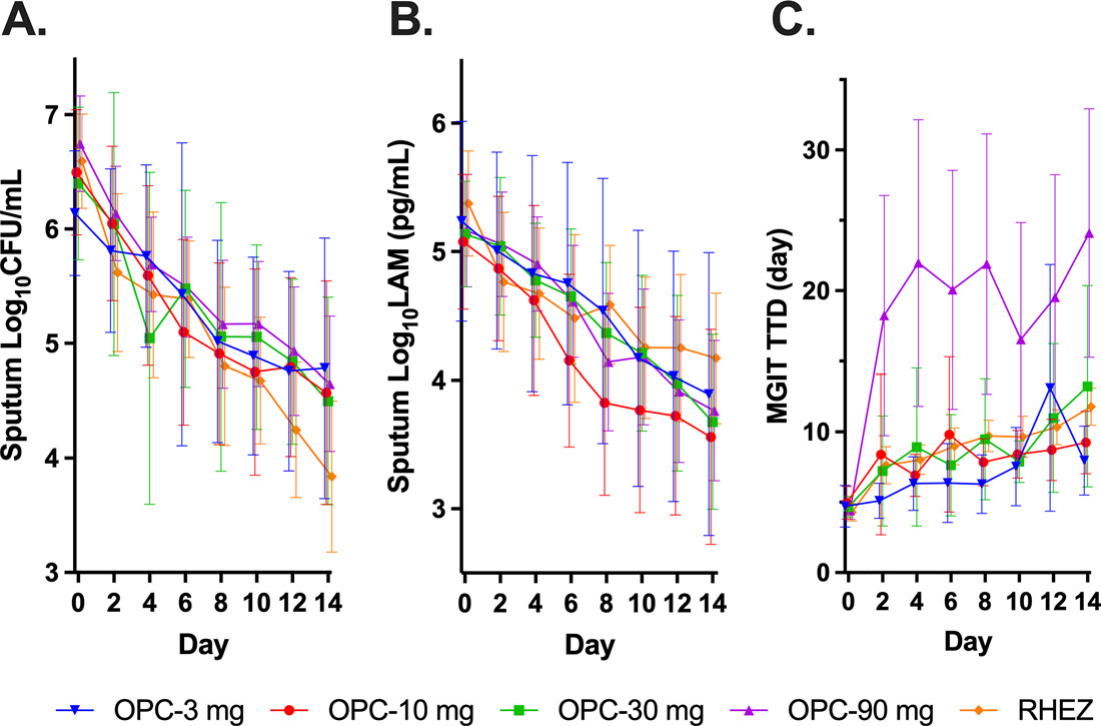
MAD/EBA study: bactericidal activity of OPC-167832. (A) Mean values (±SD) of sputum CFU/mL over the treatment period; (B) sputum lipoarabinomannan concentration over the treatment period; and (C) time to detection in mycobacteria growth indicator tubes over the treatment period.

All the tested OPC-167832 doses appeared to have significant bactericidal activity (Fig. 3). Across all dosing cohorts, OPC-167832 was 61% to 76% as active as RHEZ in reducing CFU/mL. At 14 days, changes from baseline in mean log_10_ CFU/mL were (mean ± standard deviation [SD]) −1.69 ± 0. 96 (*n* = 11), −1.93 ± 0.98 (*n* = 14), −2.12 ± 1.01 (*n* = 11), and −2.08 ± 0.75 (*n* = 13) for OPC-167832 at 3, 10, 30, and 90 mg doses, respectively, and −2.79 ± 0.96 (*n* = 15) for RHEZ. Reductions in LAM concentrations and increases in MGIT-TTD were consistent with the observed potent mycobacterial killing (Fig. 2 and Table 3). Notably, a large acceleration of the increase in TTD was observed in the OPC-167832 90 mg arm between start of treatment and day 2, which stabilized from day 3. This is currently being investigated as an artifact secondary to drug carryover. The increase of MGIT-TTD in the 30-mg arm was also more than the RHEZ arm, indicating potential drug carryover for the 30-mg arm, as well.

**TABLE 3:** Bactericidal activity as differences between values at day 14 and baseline^*a*^

Parameter	OPC-167832				RHEZ
	3 mg	10 mg	30 mg	90 mg	
log_10_CFU	–1.69 ± 1.15 (*n* = 11)	–1.93 ± 0.98 (*n* = 14)	–2.21 ± 1.01 (*n* = 10)	–2.08 ± 0.75 (*n* = 13)	–2.79 ± 0.96 (*n* = 15)
log_10_LAM	–1.35 ± 0.76 (*n* = 13)	–1.52 ± 0.79 (*n* = 11)	–1.41 ± 0.75 (*n* = 11)	–1.44 ± 0.67 (*n* = 14)	–1.19 ± 0.75 (*n* = 15)
MGIT-TTD	3.33 ± 2.74 (*n* = 13)	4.48 ± 2.88 (*n* = 14)	9.54 ± 13.63 (*n* = 11)	18.15 ± 15.06 (*n* = 13)	7.44 ± 1.65 (*n* = 15)

a Values shown are mean ± SD; a negative value indicates reduction from the baseline value. LAM, lipoarabinomannan; MGIT, Mycobacteria Growth Indicator Tube; *n*, number of samples available for assessment; TTD, time to detection.

To further explore the applicability of LAM as a marker of treatment response, the relationship between LAM concentrations in the raw sputum (i.e., the sputum used for CFU counting) and the CFU/mL count on 7H11 medium was analyzed. Results indicated a strong correlation between log_10_ LAM concentration and log_10_ CFU/mL (Pearson correlation coefficient of 0.8631; Fig. S1).

### Safety.

In the SAD study, the most commonly reported TEAEs in the combined OPC-167832 groups were headache (3/36 [8.3%]), constipation (2/36 [5.6%]), and back pain (2/36 [5.6%]) (Table S5). All TEAEs were mild and none led to study discontinuation; no dose-limiting toxicities were reported in any of the 6 cohorts, indicating the maximum tolerated single dose for OPC-167832 was not reached by 480 mg. TEAEs assessed by the investigator as potentially related to OPC-167832 treatment included 2 cases of mild constipation (1 case each in the 60-mg and 480-mg cohorts) and 1 case of mild paresthesia in the 240-mg cohort. In addition, 1 subject had mild lacrimation increase, photophobia, eye irritation, and headache that was potentially related to OPC-167832 treatment, as well as mild conjunctivitis that was considered unrelated to study drug. No serious TEAEs were reported. Alterations in physical examination findings, vital signs, and clinical laboratory results were minimal in all dosing cohorts. Abnormal electrocardiogram results were rare and not considered to be clinically significant.

In the food effects study, the most common TEAEs in the combined treatment sequences were alanine transaminase (ALT) increase (2/12 [16.7%] participants) and pruritus (2/12 [16.7%] participants) (Table S6). ALT elevation and pruritus events occurred in separate participants. All TEAEs were considered mild, except for 1 case of moderate transient blood creatine phosphokinase increase. TEAEs assessed by the investigator as potentially related to OPC-167832 treatment included mild arthralgia and ecchymosis after a standard meal in one subject; mild headache, pruritus, and xerosis after fasting in one subject; mild eye irritation after fasting in one subject; mild paraesthesia and pollakiuria after fasting and mild alopecia after a standard meal in one subject; and two events of mild ALT increase in one subject, one event occurring after a standard meal and the other after a high-fat meal, which were not thought to be clinically significant. No serious TEAEs were reported. Alterations in physical examination findings and vital signs were minimal in both treatment sequences. No consistent, clinically significant ECG changes were observed following OPC-167832 administration.

In the MAD/EBA study, the most common TEAEs in the combined OPC-167832 groups were headache (12/59 [20.3%]) and pruritus (11/59 [18.6%] participants) (Table 4). All TEAEs were considered mild, except for 1 case of serious hemoptysis in the 3-mg cohort, which was judged to be related to the underlying pulmonary TB rather than to study drug. No other serious TEAEs were reported. Alterations in physical examination findings, vital signs, ECGs, and clinical laboratory results were minimal in all dosing cohorts.

**TABLE 4:** Incidence of all treatment-emergent adverse events in the MAD/EBA study

System organ class preferred term	OPC-167832, *n* (%)					RHEZ^*a*^
	3 mg (*n* = 14)	10 mg (*n* = 14)	30 mg (*n* = 14)	90 mg (*n* = 17)	Total (*n* = 59)	(*n* = 16)
Headache	3 (21.4)	3 (21.4)	4 (28.6)	2 (11.8)	12 (20.3)	2 (12.5)
Pruritus	2 (14.3)	1 (7.1)	5 (35.7)	3 (17.6)	11 (18.6)	6 (37.5)
Conjunctival irritation	1 (7.1)	0 (0.0)	1 (7.1)	2 (11.8)	4 (6.8)	0 (0.0)
Vomiting	0 (0.0)	2 (14.3)	2 (14.3)	0 (0.0)	4 (6.8)	2 (12.5)
Myalgia	0 (0.0)	1 (7.1)	0 (0.0)	3 (17.6)	4 (6.8)	1 (6.3)
Hemoptysis	1 (7.1)	1 (7.1)	1 (7.1)	1 (5.9)	4 (6.8)	3 (18.8)
Eye pain	0 (0.0)	0 (0.0)	3 (21.4)	0 (0.0)	3 (5.1)	0 (0.0)
Abdominal pain	0 (0.0)	1 (7.1)	1 (7.1)	1 (5.9)	3 (5.1)	2 (12.5)
Diarrhea	0 (0.0)	0 (0.0)	2 (14.3)	1 (5.9)	3 (5.1)	1 (6.3)
Noncardiac chest pain	0 (0.0)	3 (21.4)	0 (0.0)	0 (0.0)	3 (5.1)	0 (0.0)
Pyrexia	1 (7.1)	1 (7.1)	0 (0.0)	1 (5.9)	3 (5.1)	1 (6.3)
Arthralgia	0 (0.0)	1 (7.1)	2 (14.3)	0 (0.0)	3 (5.1)	0 (0.0)
Dizziness	1 (7.1)	1 (7.1)	1 (7.1)	0 (0.0)	3 (5.1)	1 (6.3)
Pleuritic pain	1 (7.1)	1 (7.1)	0 (0.0)	1 (5.9)	3 (5.1)	0 (0.0)
Eye pruritus	0 (0.0)	1 (7.1)	1 (7.1)	0 (0.0)	2 (3.4)	1 (6.3)
Nausea	1 (7.1)	1 (7.1)	0 (0.0)	0 (0.0)	2 (3.4)	1 (6.3)
Back pain	0 (0.0)	2 (14.3)	0 (0.0)	0 (0.0)	2 (3.4)	1 (6.3)
Dysmenorrhea	0 (0.0)	0 (0.0)	1 (7.1)	1 (5.9)	2 (3.4)	1 (6.3)
Rash pruritic	1 (7.1)	1 (7.1)	0 (0.0)	0 (0.0)	2 (3.4)	1 (6.3)
Fatigue	1 (7.1)	0 (0.0)	0 (0.0)	0 (0.0)	1 (1.7)	1 (6.3)
Dysuria	1 (7.1)	0 (0.0)	0 (0.0)	0 (0.0)	1 (1.7)	1 (6.3)
Epistaxis	1 (7.1)	0 (0.0)	0 (0.0)	0 (0.0)	1 (1.7)	1 (6.3)
Rash	0 (0.0)	0 (0.0)	1 (7.1)	0 (0.0)	1 (1.7)	1 (6.3)

a RHEZ, Rifafour e-275.

## DISCUSSION

The development of more effective and tolerable anti-TB regimens with shorter treatment durations requires new anti-TB agents (3, 6). Three recently approved drugs—delamanid (7), pretomanid (8, 9) and bedaquiline (10, 11)—have gone some way in addressing this critical need, but the availability of additional drugs with novel mechanisms is essential to ensure the emergence of more effective combination regimens. In preclinical studies, the new antimycobacterial agent OPC-167832 has shown high activity against M. tuberculosis in murine and *in vitro* model systems and favorable pharmacokinetic and toxicity profiles in animals (3). Accordingly, advancement of this novel and potentially beneficial agent into human testing was pursued. This report describes the initial phases of a clinical development program for OPC-167832, including the first in-human EBA monotherapy trials, that will also evaluate the potential of OPC-167832-containing regimens in patients with drug-susceptible and drug-resistant TB.

In general, a less than dose-proportional increase in plasma exposure was observed for OPC-167832, as demonstrated by a slope parameter value less than 1, which was likely due to a decrease in bioavailability with increasing dose (Fig. 2). In healthy participants who received single oral doses of OPC-167832 (30 to 480 mg), maximum plasma concentrations occurred 2.5 to 3.5 h after administration and elimination half-life ranged from 19 to 30 h, supporting a simple once-daily oral dosing regimen. The mean AUC_0–inf_ for the lowest tested dose (30 mg) was 2,392 h•ng/mL, which exceeded the preclinical minimum effective AUC value. By chance, the SAD and food-effects studies only enrolled healthy male subjects, which was attributable to the exclusion during screening of many females of childbearing potential. The resulting male bias was a limitation of the phase I study, but it should be noted that no gender-specific difference in OPC-167832 exposure was observed in the MAD study (data not shown). For multiple ascending doses from 3 to 90 mg in participants with drug-susceptible TB, the pharmacokinetic characteristics were similar to healthy subjects, although with a slightly shorter half-life of 14 to 22 h. The moderate accumulation was observed with once-daily dosing, and apparent steady state was reached by day 14.

In preclinical studies, OPC-167832 was characterized by high bactericidal activity (3). In a murine TB model, the AUC_0–24h_ needed to achieve 80% of the maximum bactericidal activity (EC_80_) was identified to be 2,033 h•ng/mL (Otsuka Pharmaceuticals, data on file). This target exposure, in combination with PK results from the SAD study, guided our selection of a starting OPC-167832 dose of 10 mg in the MAD/EBA trial. Subsequent dose escalation demonstrated similar EBA in the 10-, 30-, and 90-mg dose cohorts, as judged by decline in sputum LAM concentration and CFU/mL counts, and the fourth cohort was therefore modified to receive a 3-mg dose, rather than the originally intended 270-mg dose. The efficacy results from the four tested doses (Fig. 3), considered in parallel with the safety profile (see below), provided support for selecting a 30-mg dose for future combination studies.

Sputum LAM has been proposed to be a potential biomarker to quantify bacterial load during TB treatment (5), and data in Fig. S1 demonstrated strong correlations of log_10_ CFU and log_10_ LAM. In this study, we used sputum LAM as a biomarker to guide dose escalation, as LAM results were obtained in near real-time compared to CFU results, which required several weeks to finalize. This approach worked well, allowing the escalation/change of dose arms with minimal delays. Of note, and as shown in Table 3, the EBA based on log_10_ LAM in the RHEZ arm is less than those from the OPC-167832 arms. This is in contrast with the EBAs based on log_10_ CFU. Thus, the value of sputum LAM as a biomarker will need further validation in future clinical trials (5).

With no reported dose-limiting toxicities, tolerability of OPC-167832 was confirmed in healthy participants at single doses up to 480 mg and in participants with TB for multiple doses up to 90 mg. Nearly all reported TEAEs in the three studies were mild in intensity and self-limiting, with only headache and pruritus occurring among the most commonly reported events in at least two of the studies. Alterations in physical examination findings, vital signs, clinical laboratory results, and ECGs were minimal in all participants (including no evidence of clinically significant QTc prolongation). Overall, these favorable tolerability and safety findings for OPC-167832 were consistent with the safety profiles of other carbostyril-based drugs (12).

The promising safety profile and potent bactericidal activity observed with monotherapy in the current MAD/EBA study encourages further clinical development of OPC-167832, including evaluation of the effect of regimens containing OPC-167832 in sputum culture conversion and relapse-free cure. A second stage of the phase I/IIa study, ongoing at the time of this report, is comparing three combination drug regimens—OPC-167832/delamanid, OPC-167832/bedaquiline and OPC-167832/delamanid/bedaquiline—in participants with pulmonary TB. OPC-167832 is being administered in the above combinations at once-daily doses of 30 mg, and results from this trial are forthcoming.

To summarize, in these first in-human studies, OPC-167832 met expectations raised during its preclinical development by exhibiting high EBA in participants with drug-susceptible, smear-positive, pulmonary TB, as well as favorable pharmacokinetic, tolerability, and safety profiles. Future evaluation of OPC-167832-containing regimens on sputum culture conversion and relapse-free cure are warranted.

## MATERIALS AND METHODS

The phase I single ascending dose (SAD) and food effects study was conducted at one site in Toronto, Canada (INC Research Toronto, Inc.), and the phase I/IIa multiple ascending dose/early bactericidal activity (MAD/EBA) study (ClinicalTrial.gov identifier: NCT03678688) was conducted at two sites in South Africa (University of Cape Town Lung Institute [Pty.] Ltd., Cape Town, South Africa and TASK Clinical Research Centre, Cape Town, South Africa). Both trials were sponsored by Otsuka America Pharmaceutical Development & Commercialization, Inc. (Rockville, MD, USA) and complied with International Conference on Harmonization and Good Clinical Practice guidelines for conducting, recording, and reporting clinical trials and for archiving essential documents, as well as with current regulatory and ethical guidelines at the study sites. Written consent was obtained from each participant. The informed consent form, protocol and amendments for the study were approved by the institutional review board or independent ethics committee at each trial site.

### Participants.

The SAD and food effects study enrolled healthy adult participants (males and females of nonchildbearing potential) from 18 to 45 years of age. Health status was determined by medical history, physical examination, electrocardiogram (ECG), serum/urine clinical chemistry, hematology, and serology tests. Participants were excluded if they had a body mass index (BMI) outside the range of 18.0 to 32.0 kg/m^2^; a history of any clinically significant abnormalities, including hemorrhagic tendencies; alcohol and/or substance abuse; tobacco use or exposure to second-hand smoke within the prior 2 months; exposure to prescription medications, over-the-counter medications, herbal medications, or vitamin supplements within the prior 14 days; or exposure to antibiotics and/or substances known to stimulate hepatic microsomal enzymes within the prior 30 days. Participants were also excluded if they had blood pressure outside the range of 100/50 to 140/90 mm Hg, pulse rate outside the range of 50 to 90 bpm, and a QTcF (QT interval corrected for heart rate by Fridericia's formula) greater than 450 msec.

The MAD/EBA study enrolled male and female adults from 18 to 64 years of age with newly diagnosed (within the prior 3 months) rifampicin- and isoniazid-susceptible pulmonary TB. At screening, participants had to exhibit acid-fast bacilli (≥1+) in sputum per microscopic observation using WHO/IUATDL methodology (13), as well as susceptibility to rifampicin and isoniazid. Participants were excluded if they had a BMI outside the range of 16.0 to 32.0 kg/m^2^; clinical evidence of severe extrapulmonary TB, pulmonary silicosis, lung fibrosis or other severe lung condition; renal or hepatic impairment; current and/or history of substance and/or alcohol abuse; history of hepatitis; a positive HIV test; treatment for M. tuberculosis within the past 3 years; or treatment with a drug active against M. tuberculosis within the prior 3 months. Participants were also excluded if they had any of the following cardiac anomalies: QTcF greater than 450 msec; atrioventricular block II or III; bifascicular block; or current and/or history of clinically significant ventricular arrhythmias.

### Study design.

**(i) Phase I SAD and food effects study.** The phase I study consisted of two parts. Part 1 was a randomized, double-blind, placebo-controlled SAD study in healthy adults with 6 dosing cohorts. Each cohort included 8 participants who were randomized 3:1 to receive a single oral dose of OPC-167832 (*n* = 6) or matching placebo (*n* = 2). Cohorts were assessed sequentially at OPC-167832 doses of 30, 60, 90, 120, 240, and 480 mg, with enrollment into a new cohort occurring only after a review of safety and initial pharmacokinetics (PK) data from the prior cohort. The starting dose of OPC-167832 was selected based on expected exposure relative to it having no observed adverse effects in nonclinical toxicological studies and on US FDA guidance for calculating maximum human starting dose (14). All doses were administered following a standard breakfast, and participants remained in-house at the trial site for 8 days for assessments.

Part 2 of the phase I study, a food effects study, was a randomized, open-label, crossover trial with two parallel treatment sequences in healthy adult participants. Twelve participants each received 3 single 60-mg oral doses of OPC-167832 separated by 14-day washout periods. Six of the participants were randomized to receive the 3 doses according to the following crossover treatment sequence: first, after a standard meal (332 cal, with 12% from fat); second, in a fasted state; and third, after a high-fat meal (800 to 1,000 cal, with 50% from fat) (15). The remaining 6 received the 3 doses according to the following sequence: first, in a fasted state; second, after a high-fat meal; and third, after a standard meal. In the fasted state, study treatments were administered in the morning following an overnight fast lasting at least 10 h, and no food was allowed for at least 4 h postdose. In the fed state, study treatments were administered within 5 min after completion of a standard or high-fat meal. Participants remained at the trial site until the morning of day 8 of each treatment period for assessments and were then discharged to complete the 14-day washout periods.

**(ii) Phase I/IIa MAD/EBA study.** The phase I/IIa MAD/EBA study consisted of two stages. Stage 1 was a multicohort, active-controlled, randomized, open-label, multiple ascending dose study of oral OPC-167832 in HIV-negative adults with smear-positive, rifampicin- and isoniazid-susceptible pulmonary TB. Stage 1 had four treatment cohorts. In each cohort, participants were randomly assigned to receive 14 days of either once-daily oral OPC-167832 (target, *n* = 14 per cohort) or local standard of care (weight-banded Rifafour e-275 [RHEZ]; target, *n* = 4 per cohort) following a standard meal. All study drugs were administered within 5 to 10 min after completing a meal.

The first cohort was treated with OPC-167832 at a dosage of 10 mg; the 10-mg dose was predicted to produce an area under the concentration-time curve (AUC) achieving 80% of the maximum bactericidal activity in humans, based on pharmacokinetic-pharmacodynamic analyses in a mouse TB model (unpublished data) and PK results from the SAD study. Safety, tolerability, PK, and LAM-ELISA data (see next section [Assessments]) from each cohort were reviewed by the trial review team at the end of each cohort to determine if the dose level for the next cohort would be escalated. Notably, the decline in sputum LAM concentration allowed for a real-time proxy for EBA, given its correlation with culture-based methods in previous studies (16). Subsequent cohorts were planned at doses of 30, 90, and 270 mg. However, similar bactericidal activities, based mainly on decline in sputum LAM concentrations, and later confirmed by decline in CFU/mL, were observed in the 10-, 30-, and 90-mg dose cohorts. Therefore, the 270-mg dose was dropped, and a 3-mg cohort was added in hopes of defining a better exposure-response relationship. Participants remained at the trial site until day 20 for final collection of safety, tolerability, pharmacokinetic, and EBA assessments (see Assessments section). Following study treatment, participants were referred to local clinics to receive therapy for pulmonary TB according to local standard of care.

Stage 2 of the MAD/EBA study, ongoing at the time of this report, will evaluate the safety and tolerability of multiple oral doses of OPC-167832 (30 mg QD) in combination with delamanid and/or bedaquiline compared to RHEZ alone in a population of patients with TB that is similar to Stage 1. Results from Stage 2 will be presented in a future publication.

### Assessments.

**(i) Pharmacokinetic sampling and bioanalytical methods.** In the SAD and food effects studies, pharmacokinetic blood samples were drawn on day 1 (within 2 h prior to dosing and 0.5, 1, 2, 3, 4, 5, 6, 8, 12, and 16 h postdose), day 2 (24 and 36 h postdose), day 3 (48 h postdose), day 4 (72 h postdose), day 5 (96 h postdose), day 6 (120 h postdose), and day 8 (168 h postdose), or at early termination for each study treatment.

In the MAD/EBA study, pharmacokinetic blood samples were collected on day 1 (predose and 0.5, 1, 2, 3, 4, 5, 6, 8, and 12 h postdose), day 2 (24 h after the day 1 dose), days 12 and 13 (predose), day 14 (predose and 0.5, 1, 2, 3, 4, 5, 6, 8, and 12 h postdose), day 15 (24 and 36 h after the day 14 dose), days 16, 17, 18, 19, and 20 (48, 72, 96, 120, and 144 h after the last dose on day 14 dose, respectively).

For the analysis of OPC-167832, 4-mL blood samples were collected into tubes containing dipotassium ethylenediaminetetraacetic acid (K_2_EDTA) to isolate plasma for bioanalysis. OPC-167832 levels in blood samples from both the SAD and MAD/EBA studies were assessed by a validated high-performance liquid chromatography/tandem mass spectrometry assay, with a lower limit of quantitation of 1 ng/mL. Both OPC-167832 and the internal standard OPC-177169 were extracted by protein precipitation from 100 μL of blood plasma containing K_2_EDTA as an anticoagulant. Analytes were chromatographically separated by reverse-phase HPLC on an Acquity BEH C18 1.7 μm, 2.1 × 50-mm column and detected by tandem mass spectrometry in positive ion mode using multiple reaction monitoring. Calibration standards prepared in plasma and processed by the same procedure as the samples were used to extrapolate the concentrations of the analyte by weighted (1/x^2^) linear regression of peak area ratios of analyte-to-internal standard over a range of 1 to 500 ng/mL. Quality control samples at 4 concentrations were assayed to monitor analytical performance. Acceptable accuracy, precision, linearity, and specificity were demonstrated. Stability experiments showed the analytes were stable under the experimental conditions of the assay.

**(ii) Bactericidal activity.** In the MAD/EBA study, overnight sputum samples were collected on two pretreatment days and on treatment days 2, 4, 6, 8, 10, 12, and 14 and subsequently analyzed for quantitative changes over time in the following parameters: CFU/mL of sputum on 7H11 culture medium; LAM concentration, as assessed by a TB LAM-ELISA (Otsuka Pharmaceutical Co., Ltd., Tokyo, Japan) (5); and time to detection/positive (TTD), as assessed in the Bactec mycobacteria growth indicator tube (MGIT) 960 automated mycobacterial detection system (Becton, Dickinson, NJ, USA). TTD (in days) was defined as the time from inoculation to positivity detected by the MGIT machine. EBA was measured by the change at the end of the 14-day treatment period from baseline in log_10_ CFU/mL, log_10_ LAM concentration (pg/mL), and MGIT TTD.

Standardized protocols for sputum processing and analysis were followed. Briefly, sputum samples collected overnight were homogenized for 30 min by magnetic stirring and a 400-μL raw sputum aliquot was removed for LAM-ELISA, according to published methodologies (5). Up to 10 mL sputum was then digested for 20 min with an equal volume of 10% dithiothreitol (Thermo Fisher Scientific, ON, Canada). Next, 1 mL of digested sputum was used to prepare a range of 10-fold dilutions from 10 to 10 exp 0, and 100 μL of each dilution was plated in quadruplicate on 7H11 agar plates containing 200 units/mL polymixin B, 10 μg/mL amphotericin B, 50 μg/mL carbenicillin and 20 μg/mL trimethoprim. CFU/mL was determined after up to 6 weeks incubation at 37°C, using the dilution that generally yielded counts between 20 to 200 colonies per plate.

Five milliliters of digested sputum was also processed using the NAC-PAC Red system (omitting the NALC) (Alph-Tec Systems, Inc., WA, USA) for 20 min prior to centrifugation and pellet resuspension to a final volume of 2 mL. An additional 400-μL aliquot was removed from the pellet for further LAM ELISA testing, and 500 μL was inoculated into a MGIT tube supplemented with OADC and PANTA (Becton, Dickinson, NJ, USA). The TTD from the MGIT instrument was only valid for pure positive MTB cultures, confirmed using Ziehl-Neelsen staining, blood agar culture, and MPT64 identification (MGIT TBc Identification Test, Becton, Dickinson, NJ, USA).

**(iii) Safety.** Safety was assessed in the SAD and MAD/EBA studies by treatment-emergent adverse events (TEAEs) (including severity, seriousness, and relationship to study drug), physical examinations, vital signs, clinical laboratory results, and electrocardiograms (ECGs). In the SAD and food effects studies, ECGs were recorded 0.75 and 0.5 h before and 0.5, 1, 2, 3, 4, 5, 6, 8, 10, 12, 16, and 24 h after each dose of study treatment. In the MAD/EBA study, ECGs were collected at screening, pretreatment, day 7 (predose) and day 14 (predose).

### Statistics, pharmacokinetic and pharmacokinetic/pharmacodynamic analysis.

Outcomes were summarized for each treatment group and for the entire population using descriptive statistics. Formal sample size calculations were not performed in either study. Pharmacokinetic parameters were analyzed by noncompartmental methods using Phoenix WinNonlin (Pharsight Corporation, version 6.4) and SAS version 9.4 (SAS Institute Inc., Cary, NC, USA). A power model was used to characterize dose proportionality of the log-transformed pharmacokinetic parameters in the single ascending dose range from 30 to 480 mg. EBA was calculated (see equation below) as the slope of the change from baseline (i.e., the mean of the values from day 2 and day 1 when both values were present; if only one value was present, it was used as baseline) to the end of the 14-day treatment from baseline in log_10_ CFU (CFU/mL), log_10_ LAM (pg/mL), and MGIT TTD (days) using SAS version 9.4 (SAS Institute Inc., Cary, NC, USA). Average EBA for each treatment and standard deviations are presented. The correlation of log_10_ CFU and log_10_ LAM was also analyzed using the SAS version 9.4 (SAS Institute Inc., Cary, NC, USA).

$$\mathrm{EB}A_{x-y,i}=\frac{\mathrm{Lo}g_{10}\;\mathrm{valu}e_{x,i}-\mathrm{Lo}g_{10}\;v\mathrm{alu}e_{y,i}}{y-x}$$
