# Supplementary material for: Phase I Single Ascending Dose and Food Effect Study in Healthy Adults and Phase I/IIa Multiple Ascending Dose Study in Patients with Pulmonary Tuberculosis to Assess Pharmacokinetics, Bactericidal Activity, Tolerability, and Safety of OPC-167832
Source: Antimicrob Agents Chemother. 2023 May 23;67(6):e01477-22. doi: 10.1128/aac.01477-22 (PMC10269160; doi:10.1128/aac.01477-22)

SUPPLEMENTAL MATERIAL FOR:

Phase I Single Ascending Dose and Food Effect Study in Healthy Adults and Phase I/IIa Multiple Ascending Dose Study in Patients with Pulmonary Tuberculosis to Assess Pharmacokinetics, Bactericidal Activity, Tolerability and Safety of OPC-167832

Rodney Dawson [1], Andreas H. Diacon [2], Kim Narunsky [1], Veronique R. De Jager [2], Kelly W. Stinson [3], Xiaoyan Zhang [4], Yongge Liu [4], Jeffrey Hafkin [4]

[1] Division of Pulmonology, Department of Medicine, University of Cape Town and University of Cape Town Lung Institute, Cape Town, South Africa.

[2] TASK Applied Science, Cape Town, South Africa.

[3] Cultura, LLC, Decatur, GA, USA.

[4] Otsuka Pharmaceutical Development & Commercialization, Inc, Rockville, MD, USA.

SUPPLEMENTAL TABLES

Table S1. SAD Study: Demographics and Baseline Characteristics.

	OPC-167832						Placebo	Total
	**30 mg (N=6)**	**60 mg (N=6)**	**90 mg (N=6)**	**120 mg (N=6)**	**240 mg (N=6)**	**480 mg (N=6)**	**Placebo (N=12)**	**Total (N=48)**
Male, n (%)	6 (100)	6 (100)	6 (100)	6 (100)	6 (100)	6 (100)	12 (100)	48 (100)
Race, n (%)								
White	5 (83.3)	3 (50.0)	4 (66.7)	5 (83.3)	3 (50.0)	2 (33.3)	8 (66.7)	30 (62.5)
Black	0	2 (33.3)	2 (33.3)	0	2 (33.3)	0	4 (33.3)	10 (20.8)
Asian	0	0	0	0	0	4 (66.7)	0	4 (8.3)
Other	1 (16.7)	1 (16.7)	0	1 (16.7)	1 (16.7)	0	0	4 (8.3)
Ethnicity, n (%)								
Hispanic/Latino	1 (16.7)	4 (66.7)	0	2 (33.3)	0	0	2 (16.7)	9 (18.8)
Not Hispanic/Latino	5 (83.3)	2 (33.3)	6 (100)	4 (66.7)	6 (100)	6 (100)	10 (83.3)	39 (81.3)
Age, years								
Mean (SD)	39.5 (5.21)	29.8 (7.99)	39.5 (4.72)	38.2 (4.49)	27.2 (6.49)	34.7 (6.77)	33.7 (7.27)	34.5 (7.34)
Range	30–45	22–43	34–45	30–43	20–38	27–43	24–44	20–45
BMI, kg/m^2								
Mean (SD)	24.48 (3.802)	21.58 (2.156)	25.85 (3.436)	25.88 (2.122)	25.83 (3.570)	25.55 (3.213)	25.72 (3.072)	25.08 (3.225)
Range	19.8–29.1	19.5–25.5	21.2–28.5	22.1–28.3	20.1–30.9	19.8–28.4	20.5–29.2	19.5–30.9

BMI, body mass index (assessed at screening visit).

Table S2. Food Effects Study: Demographics and Baseline Characteristics.

	Standard/Fasted/ High-Fat Meal (N=6)	Fasted/High-Fat Meal/ Standard (N=6)	Total (N=12)
Male, n (%)	6 (100)	6 (100)	12 (100)
Race, n (%)			
White	4 (66.7)	4 (66.7)	8 (66.7)
Black or African American	0	2 (33.3)	2 (16.7)
Asian	2 (33.3)	0	2 (16.7)
Ethnicity, N (%)			
Hispanic/Latino	3 (50.0)	2 (33.3)	5 (41.7)
Not Hispanic/Latino	3 (50.0)	4 (66.7)	7 (58.3)
Age, years			
Mean (SD)	29.3 (7.66)	31.8 (7.73)	30.6 (7.45)
Range	22–44	22–43	22–44
BMI, kg/m^2			
Mean (SD)	25.53 (3.553)	26.97 (2.715)	26.25 (3.106)
Range	20.7–29.2	24.4–31.6	20.7–31.6

BMI, body mass index (assessed at screening visit).

Table S3. MAD/EBA Study: Demographics and Baseline Characteristics.

	OPC-167832					
	OPC-3 mg (N=14)	OPC-10 mg (N=14)	OPC-30 mg (N=14)	OPC-90 mg (N=17)	RHEZ (N=17)	Total (N=76)
Age, mean years (SD)	37.2 (15.59)	31.7 (14.63)	34.9 (12.77)	36.2 (13.26)	33.4 (10.89)	34.7 (13.19)
Height, mean cm (SD)	168.4 (5.91)	169.0 (9.27)	161.3 (8.34)	164.1 (7.33)	165.8 (7.81)	165.7 (8.07)
Weight, mean kg (SD)	55.54 (8.295)	60.95 (10.061)	53.90 (7.741)	52.59 (9.754)	54.91 (6.756)	55.43 (8.827)
Body mass index, mean kg/m^2 (SD)	19.67 (2.650)	21.38 (3.212)	20.63 (2.774)	19.50 (2.269)	19.96 (1.880)	20.19 (2.581)
Male, n (%)	10 (71.4)	10 (71.4)	6 (42.9)	9 (52.9)	12 (70.6)	47 (61.8)
Race, n (%)						
White		0	0	1 (5.9)	0	1 (1.3)
Black	6 (42.9)	8 (57.1)	7 (50.0)	11 (64.7)	8 (47.1)	40 (52.6)
Mixed Race or Other	8 (57.1)	6 (42.9)	7 (50.0)	5 (29.4)	9 (52.9)	35 (46.1)
Ethnicity, n (%)						
Hispanic or Latino	0	0	0	0	0	0
Not Hispanic or Latino	14 (100.0)	14 (100.0)	12 (85.7)	17 (100.0)	15 (88.2)	72 (94.7)
Unknown	0	0	2 (14.3)	0	2 (11.8)	4 (5.3)
Sputum CFU/mL, mean log_{10} (SD) [n] [a]	6.1 (1.0) [14]	6.5 (1.0) [14]	6.5 (1.1) [11]	6.8 (0.8) [14]	6.6 (0.9) [15]	
Sputum LAM pg/mL, mean log_{10} (SD) [n] [a]	5.2 (1.4) [14]	5.1 (0.9) [14]	5.3 (0.7) [11]	5.2 (0.8) [14]	5.4 (0.8) [15]	
MGIT-TTD, mean days (SD) [n] [a]	4.7 (2.6) [14]	5.0 (2.0) [14]	4.5 (1.4) [11]	4.4 (1.1) [13]	4.2 (1.3) [15]	

CFU, colony-forming unit; LAM, lipoarabinomannan; MGIT, Mycobacteria Growth Indicator Tube; n, number of participants or available samples; RHEZ, Rifafour e-275; SD, standard deviation; TTD, time to detection.

a. Average of day −2 and day −1 prior to the first dose.

Table S4. Food Effects on OPC-167832 Pharmacokinetic Parameters. Values are mean ± SD, unless noted.

Parameter, unit	Fasted (N=11)	Standard Meal (N=10)	High-fat Meal (N=10)
C_{max}, ng/mL	125 ± 35.8	193 ± 57	237 ± 72.2
t_{max}, h (median [range])	2.00 (1.00, 4.00)	3.00 (2.00, 5.00)	3.50 (2.00, 5.03)
AUC_{last} h•ng/mL	2921 ± 1199	3560 ± 1401	4023 ± 1335
$AUC_{0–inf}$, h•ng/mL	2983 ± 1196	3742 ±1453	4112 ± 1365
$t_{½}$, h	20.6 ± 6.92	19.3 ± 7.74	21.4 ± 10.4
CL/F, mL/min	390 ± 159	310 ± 130	269 ± 86.2
CL/F/BW, mL/min/kg	4.78 ± 1.71	3.90 ± 1.28	3.29 ± 0.88

$AUC_{0–inf}$, area under the concentration-time curve from zero to infinity; $AUC_{0–24h}$, area under the concentration-time curve from zero to 24 hours postdose; BW, body weight; CL/F, apparent clearance; C_{max}, maximum (peak) plasma concentration; SD, standard deviation; $t_{1/2}$, terminal-phase elimination half-life; t_{max}, time to maximum (peak) plasma concentration.

Table S5. SAD Study: Incidence of All Treatment-Emergent Adverse Events.

System Organ Class Preferred Term [a]	Placebo (N=12)	OPC-167832 30 mg (N=6)	60 mg (N=6)	90 mg (N=6)	120 mg (N=6)	240 mg (N=6)	480 mg (N=6)	Total (N=36)
Headache	0	0	0	1 (16.7)	1 (16.7)	0	1 (16.7)	3 (8.3)
Constipation	0	0	1 (16.7)	0	0	0	1 (16.7)	2 (5.6)
Back pain	0	0	0	1 (16.7)	0	0	1 (16.7)	2 (5.6)
Cerumen impaction	0	0	0	1 (16.7)	0	0	0	1 (2.8)
Ear discomfort	0	0	0	1 (16.7)	0	0	0	1 (2.8)
Eye irritation	0	0	0	1 (16.7)	0	0	0	1 (2.8)
Lacrimation increased	0	0	0	1 (16.7)	0	0	0	1 (2.8)
Photophobia	0	0	0	1 (16.7)	0	0	0	1 (2.8)
Abdominal discomfort	0	0	0	1 (16.7)	0	0	0	1 (2.8)
Salivary hypersecretion	0	0	0	1 (16.7)	0	0	0	1 (2.8)
Catheter site hematoma	0	0	0	0	0	0	1 (16.7)	1 (2.8)
Peripheral swelling	0	0	0	1 (16.7)	0	0	0	1 (2.8)
Vessel puncture site hemorrhage	0	0	0	1 (16.7)	0	0	0	1 (2.8)
Conjunctivitis	0	0	0	1 (16.7)	0	0	0	1 (2.8)
Decreased appetite	0	0	0	1 (16.7)	0	0	0	1 (2.8)
Paresthesia	0	0	0	0	0	1 (16.7)	0	1 (2.8)
Erythema	0	0	0	1 (16.7)	0	0	0	1 (2.8)
Pruritus	0	0	0	1 (16.7)	0	0	0	1 (2.8)
Muscle tightness	0	0	0	0	0	0	1 (16.7)	1 (2.8)
Dysphonia	0	0	0	0	0	1 (16.7)	0	1 (2.8)
Epistaxis	0	0	0	0	0	1 (16.7)	0	1 (2.8)
Oropharyngeal pain	1 (8.3)	0	0	0	0	1 (16.7)	0	1 (2.8)

System Organ Class<br>Preferred Term [a]	Placebo<br>(N=12)	OPC-167832						
		30 mg<br>(N=6)	60 mg<br>(N=6)	90 mg<br>(N=6)	120 mg<br>(N=6)	240 mg<br>(N=6)	480 mg<br>(N=6)	Total<br>(N=36)
Cough	1 (8.3)	0	0	0	0	0	0	0
Nasal congestion	1 (8.3)	0	0	0	0	0	0	0

Participants were counted once, per term, for the most severe of multiple occurrences of a specific MedDRA preferred term.

a. Participants with adverse events in multiple System Organ Classes under the same dose group were counted only once towards the total row.

Table S6. Food Effects Study: Incidence of all Treatment-Emergent Adverse Events.

System Organ Class<br>Preferred Term, n (%)	Standard Meal<br>(N=10)	Fasted<br>(N=11)	High-Fat Meal<br>(N=10)	Total<br>(N=12) [a]
Increased alanine aminotransferase	1 (10.0) [b]	1 (9.1) [c]	1 (10.0) [b]	2 (16.7)
Pruritus	0	2 (18.2)	0	2 (16.7)
Eye discharge	1 (10.0)	0	0	1 (8.3)
Eye irritation	0	1 (9.1)	0	1 (8.3)
Xerosis	0	1 (9.1)	0	1 (8.3)
Skin abrasion	0	1 (9.1)	0	1 (8.3)
Increased aspartate aminotransferase	0	1 (9.1)	0	1 (8.3)
Increased blood creatine phosphokinase	0	1 (9.1)	0	1 (8.3)
Increased blood lactate dehydrogenase	0	1 (9.1)	0	1 (8.3)
Arthralgia	1 (10.0)	0	0	1 (8.3)
Pain in extremity	1 (10.0)	0	0	1 (8.3)
Headache	0	1 (9.1)	0	1 (8.3)
Paresthesia	0	1 (9.1)	0	1 (8.3)
Pollakiuria	0	1 (9.1)	0	1 (8.3)
Rhinitis allergic	0	1 (9.1)	1 (10.0)	1 (8.3)
Alopecia	1 (10.0)	0	0	1 (8.3)
Ecchymosis	1 (10.0)	0	0	1 (8.3)

a. A subject with AEs in multiple periods was counted only once for total column.
b. Events occurred in the same subject on Day 7/Period 1 and on Day 7/Period 3 (standard, then fasted, then high-fat sequence).
c. Event occurred in a second subject on Day 15/Period 1 (fasted, then high-fat, then standard sequence).

SUPPLEMENTAL FIGURES

Figure S1. MAD/EBA Study: Log_{10}LAM Concentration Versus Log_{10}CFU/mL Following Treatment With Once-Daily OPC-167832 in Participants with Drug-Susceptible Pulmonary TB.

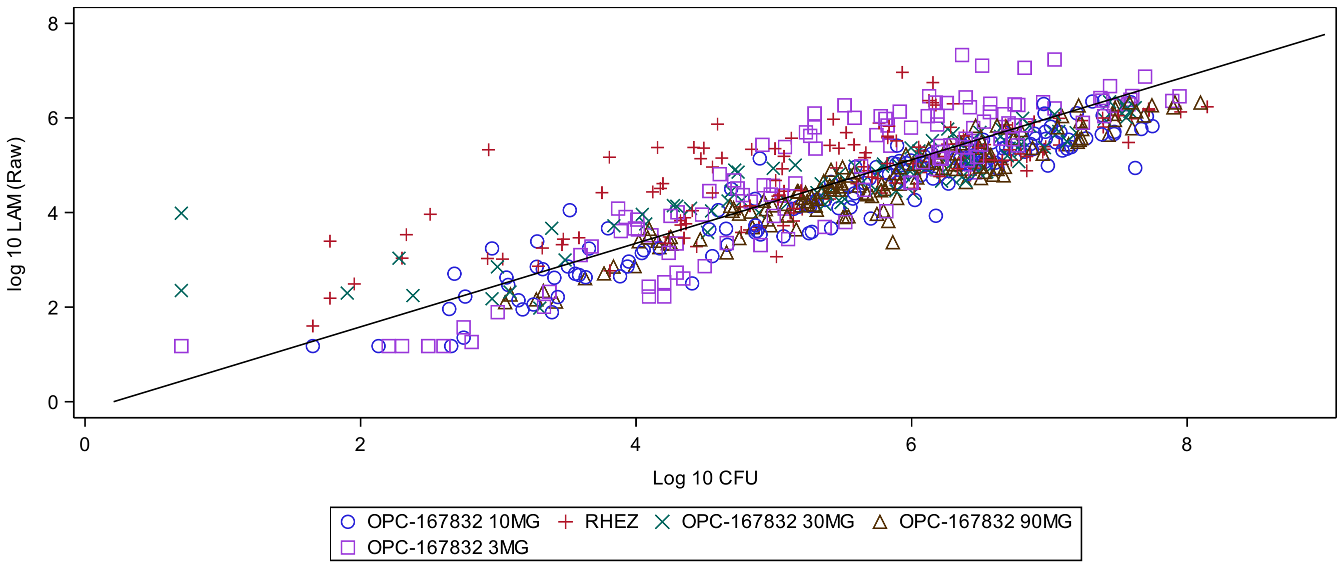

Supplement: Supplemental file 1 — Supplemental material. Download aac.01477-22-s0001.pdf, PDF file, 0.3 MB [file aac.01477-22-s0001.pdf]
